# The Role of Pre-treatment Inflammatory Biomarkers in the Prediction of an Early Response to Panitumumab in Metastatic Colorectal Cancer

**DOI:** 10.7759/cureus.24347

**Published:** 2022-04-21

**Authors:** Angioletta Lasagna, Marta Muzzana, Virginia V Ferretti, Catherine Klersy, Anna Pagani, Daniela Cicognini, Paolo Pedrazzoli, Silvia G Brugnatelli

**Affiliations:** 1 Medical Oncology, Fondazione Institute for Research, Hospitalization and Health Care (IRCCS) Policlinico San Matteo, Pavia, ITA; 2 Service of Clinical Epidemiology & Biostatistic, Fondazione Institute for Research, Hospitalization and Health Care (IRCCS) Policlinico San Matteo, Pavia, ITA

**Keywords:** skin and mucosal toxicity, serum biomarkers, predictive value, panitumumab, lmr, neutrophil-to-lymphocyte ratio (nlr)

## Abstract

Background

Systemic inflammation is a critical component of the development and progression of several types of cancer. Neutrophil-lymphocyte ratio (NLR) and lymphocyte-monocyte ratio (LMR) are simple, inexpensive, and reliable predictors of the systemic inflammatory response to the therapy in different malignant tumors, including colorectal cancer.

Methods

Metastatic colorectal cancer (mCRC) patients treated with panitumumab plus chemotherapy at first-line at the medical oncology unit of Fondazione Institute for Research, Hospitalization and Health Care (IRCCS) Policlinico San Matteo di Pavia between January 1st 2016 and February 1st 2021 were retrospectively analyzed. NLR and LMR were divided into two groups (high and low) based on the cut-off points, with the estimation of the prognostic accuracy of NLR for the early treatment response as the primary end-point of this study.

Results

The receiver operating characteristic (ROC) analysis showed a fair prognostic accuracy of NLR for early treatment response (area under the curve (AUC)=0.76, 95% CI: 0.62-0.89). A slightly lower prognostic accuracy was found for LMR (AUC=0.71, 95% CI: 0.57-0.85). In the univariable proportional hazard Cox model, no effect of NLR on PFS was found (NLR^High^ vs. NLR^Low^ HR=1.3; 95% CI: 0.7-2.4, p=0.414). Patients with higher levels of LMR showed a trend towards higher PFS (LMR^High^ vs. LMR^Low^ HR=0.4; 95% CI: 0.2-1.1, p=0.066). No association was found between NLR (or LMR) and skin toxicity.

Conclusions

NLR and LMR may be used as biomarkers of prognostic accuracy for the early treatment response in mCRC patients treated with panitumumab.

## Introduction

(both sexes, all ages) in 2020 according to GLOBOCAN 2020 data has been of 1.931.590 [1]. The prognosis of locally advanced or metastatic colorectal cancer (mCRC) with wild-type (WT) RAS has improved due to the introduction of new targeted therapies such as epidermal growth factor receptor (EGFR) inhibitors. EGFR is a molecular therapeutic target that activates various signaling pathways that regulate cell proliferation, and its pathway has a role in cyclooxygenase 2 (COX2) expression and it is related to inflammation [2]. Panitumumab is a fully human monoclonal IgG2 anti-EGFR antibody produced in a mammalian cell line (CHO) by recombinant DNA technology [3].

Systemic inflammation is a critical component of the development and progression of several types of cancer, facilitating genomic instability and angiogenesis [4]. Recent studies have demonstrated that various inflammatory markers, such as neutrophil-lymphocyte ratio (NLR) and lymphocyte-monocyte ratio (LMR), play a predictive role in the survival of different malignant tumors, including colorectal, breast, and kidney cancers [5-7]. These biomarkers are a simple, inexpensive, and reliable predictor of the systemic inflammatory response to the therapy. In particular, a recent study has showed a positive correlation between pre-treatment NLR and progression-free survival (PFS) and overall survival (OS) in patients with mCRC treated with cetuximab in the first line [8]. In contrast, in another paper, there has been no statistically significant OS difference in patients who received anti-EGFR therapy in the first line (cetuximab or panitumumab) according to NLR [9].

Currently, there are no published studies evaluating the predictive and/or prognostic role of these markers specifically for the first-line therapy with panitumumab.

Moreover, the inhibition of the EGFR in suprabasal keratinocytes and in hair follicles causes an abnormal proliferation and an inflammatory reaction with the release of chemokines and the recruitment of the lymphocyte leading to typical dermatological toxicity, i.e., palmar-plantar erythrodysesthesia [10]. Dermatologic-related reactions are experienced with nearly all patients (approximately 94%) treated with panitumumab [11]. These adverse events can delay or interrupt the active therapy and have a negative effect on the quality of life (QoL), so the optimal therapy always involves a careful balance between efficacy and safety.

To date, the predictive role of NLR and LMR in the early toxicity of panitumumab has not been investigated.

The aim of this study was to estimate the prognostic accuracy of NLR and LMR for early response and their association with disease progression and skin toxicity in mCRC patients treated at first-line with panitumumab and chemotherapy.

## Materials and methods

Study population, participants, and period

In this observational retrospective single-center cohort study, we reviewed all patients with metastatic colorectal cancer (mCRC) who had received panitumumab plus chemotherapy at first-line at the medical oncology unit of Fondazione Institute for Research, Hospitalization and Health Care (IRCCS) Policlinico San Matteo di Pavia between January 1st 2016 and February 1st 2021. All participants had received written information with details regarding the study and had provided their signed informed consent.

The study was conducted according to the Strengthening the Reporting of Observational Studies in Epidemiology (STROBE) statement for reporting observational studies [12].

Medical records were reviewed to collect the socio-demographic and clinical characteristics of the enrolled patients. Baseline characteristics, including the clinical stage, the histology, and type of chemotherapy, were retrieved. Blood parameters were collected at baseline (before the start of the treatment), including white blood cells (103/μL), neutrophils (103/μL), lymphocytes (103/μL), monocytes (103/μL), neutrophil to lymphocyte ratio (NLR), lymphocyte to monocyte ratio (LMR).

Panitumumab was administered at a dose of 6 mg/kg every two weeks with chemotherapy (oxaliplatin-based/irinotecan-based/5-FU-based). Patients having a history of infection, previous exposure to EGFR-targeting therapy, and evidence of hematology diseases and/or autoimmune diseases before initiation of chemotherapy were excluded.

NLR was defined as the ratio of absolute neutrophil and lymphocyte count within 30 days before the initiation of chemotherapy. LMR was defined as the ratio of lymphocyte and monocyte count within 30 days before the initiation of chemotherapy.

Response assessment and skin toxicities

The response was assessed every three or four treatment cycles using the revised response evaluation criteria in solid tumors version 1.1: complete response (CR), partial response (PR), stable disease (SD), and progressive disease (PD). CR was defined as the disappearance of all the target lesions, PR was defined as at least a 30% decrease in the sum of diameters of the target lesions, SD was defined as neither sufficient shrinkage to qualify for PR nor sufficient increase to qualify for PD. PD was defined as at least a 20% increase in the sum of diameters of the target lesions and/or the appearance of one or more new lesions.

The early treatment response (CR+PR = response versus SD+PD = early absence of response) is evaluated after three months from the start of the therapy with panitumumab (given every two weeks).

Progression-free survival (PFS) is measured as the time between the start of the first line of therapy with panitumumab and the disease progression or death from any cause. We have chosen PFS because several studies have shown that it is a valid surrogate for OS in colorectal cancer [13].

The definition of dermatological toxicity is based on the National Cancer Institute (NCI) Common Terminology Criteria for Adverse Events (CTCAE 5.0). A grading (severity) scale is provided for each adverse event (AE) term.

The primary end-point was the estimation of the prognostic accuracy of NLR for the early treatment response. The main secondary objective was the estimation of the prognostic accuracy of LMR for the early treatment response. Other secondary objectives were: (i) evaluation of the effect of NLR and LMR on PFS, and (ii) evaluation of the effect of NLR and LMR on early (after four weeks) onset of skin toxicity.

The study was approved by the local Ethics Committee (Comitato Etico Area Pavia) and Institutional Review Board (P-20210068912). All the subjects signed an informed written consent.

Statistical analysis

Fifty-four patients met the eligibility criteria and therefore were included in the study. With this sample size, we had a power≥80% to detect a difference of 0.2 between the area under the ROC curve (AUC) under the null hypothesis of 0.7 and an AUC under the alternative hypothesis of 0.9 using a two-sided z-test at a significance level of 0.05.

Qualitative variables were described as counts and relative frequencies of each category. Quantitative variables have been summarized with the median and the 25th-75th percentiles.

To evaluate the prognostic accuracy of NLR and LMR for treatment response (CR+PR vs. SD+PD) after three months from the start of therapy, receiver operating characteristic (ROC) analyses were performed. The area under ROC curve (AUC) with its 95% confidence interval (95%CI) is reported. In addition, the best cut-offs of NLR and LMR to predict treatment response were identified by Youden Index.

Univariable proportional hazard Cox models were performed to estimate the prognostic effect of NLR and LMR on PFS. Due to the low number of events, only bivariable models were carried out to adjust this effect. Kaplan-Meier product-limit method was used to estimate the PFS, and survival curves were plotted according to cut-offs previously identified.

Univariable logistic regression models were applied to evaluate the effect of NLR and LMR on the onset of skin toxicity, and pre-defined multivariable logistic regression models were carried out to adjust these associations.

Two-sided p-values lower than 0.05 were considered significant. Statistical analyses were performed using Stata 17 (StataCorp, College Station, Texas).

## Results

Characteristics of the study population

Fifty-four patients’ records (18 females and 36 males; median age 66, 25th-75th 60-73) were retrospectively reviewed. At the time of the data collection, 14 patients (25.9%) were still on panitumumab, while 40 were not (74.1%). All general clinical characteristics are shown in Table 1. According to the location of the tumor, 42 patients (77.8%) had a tumor in the left colon while the other 12 (22.2%) in the right colon. Primary tumor resection was performed in 34 patients (63%), and 23 (42.6%) underwent neoadjuvant/adjuvant chemotherapy. Liver metastasis occurred in 36 patients (66.7%).

**Table 1:** Patients’ characteristics NLR - neutrophil-lymphocyte ratio, LMR - lymphocyte-monocyte ratio

Sex		N	%
Female/Male	18/36		33.3 / 67.7
Age, median (25^th^-75^th^)	66 (60-73)		
Tumor site			
Right/left	12/42		22.2 / 77.8
Previous adjuvant or neoadjuvant therapy	23		42.6
Initially metastatic	28		51.9
Operated primary	34		63.0
Initial chemotherapy			
Oxaliplatin-based	43		79.7
Irinotecan-based	9		16.7
5-FU-based	1		1.8
None	1		1.8
Number of metastatic sites			
1	35		64.8
2	11		20.4
≥ 3	8		14.8
Site of metastases			
Lung	15		27.8
Liver	36		66.7
Bone	1		1.8
Lymphnodes	14		25.9
Others	15		27.8
T categories (TNM staging)			
T1	0		0
T2	0		0
T3	30		56
T4	24		44
N categories (TNM staging)			
N1	19		35
N2	35		65
NLR, median (25^th^-75^th^)	2.7 (1.5-3.5)		
LMR, median (25^th^-75^th^)	2.3 (1.7-2.8)		

Predictive value of NLR and LMR and early treatment response

The ROC analysis showed a fair prognostic accuracy of NLR for early treatment response (AUC=0.76, 95%CI: 0.62-0.89). The Youden Index identified 2.72 as the optimal cut-off for NLR to 'classify' early treatment response, with a sensitivity of 75% (95%CI: 55.1%-89.3%) and a specificity of 76.9% (95%CI: 56.4%-91.0%). According to this cut-off, 27 patients belonged to the NLR^Low^ group (NLR<2.72), and 27 belonged to the NLR^High^ group (NLR≥2.72).

A slightly lower prognostic accuracy was found for LMR (AUC=0.71, 95%CI: 0.57-0.85). The optimal cut-off for LMR according to Youden Index was 2.81 (sensitivity: 42.3%, 95%CI 23.4%-63.1%; specificity: 92.9% 95%CI: 76.5%-99.1%). According to this cut-off, 41 patients belonged to the LMR^Low^ group (LMR<2.81), and 13 belonged to the LMR^High^ group (LMR≥2.81).

NLR and LMR and progression-free survival

At the univariable proportional hazard Cox model, no effect of NLR on PFS was found (NLR^High^ vs. NLR^Low^ HR=1.3; 95%CI: 0.7-2.4, p=0.414). This result was confirmed in all bivariable models shown in Table 2.

**Table 2:** Adjusted effects of NLR and LMR on PFS (hazard ratios derived from bivariable Cox models) NLR - neutrophil-lymphocyte ratio, LMR - lymphocyte-monocyte ratio

Models	NLR^High^ vs NLR^Low^	LMR^High^ vs LMR^Low^
Adjusting for age	HR=1.5, 95%CI: 0.8-3.0 p=0.215	HR=0.4, 95%CI: 0.2-1.0 p=0.058
Adjusting for chemo oxaliplatin	HR=1.3, 95%CI: 0.7-2.5 p=0.382	HR=0.4, 95%CI: 0.2-1.0 p=0.043
Adjusting for previous adjuvant	HR=1.5, 95%CI: 0.8-2.8 p=0.252	HR=0.4, 95%CI: 0.2-0.9 p=0.028
Adjusting for initially metastatic	HR=1.4, 95%CI: 0.7-2.6 p=0.307	HR=0.4, 95%CI: 0.2-0.9 p=0.037
Adjusting for N° of metastatic sites	HR=1.1, 95%CI: 0.5-2.4 p=0.757	HR=0.5, 95%CI: 0.2-1.1 p=0.085
Adjusting for tumor site	HR=1.4, 95%CI: 0.7-2.8 p=0.288	HR=0.4, 95%CI: 0.2-1.0 p=0.054

Conversely, patients with higher levels of LMR showed a trend towards higher PFS (LMR^High^ vs. LMR^Low^ HR=0.4; 95%CI: 0.2-1.1, p=0.066). This trend was confirmed when adjusting for oxaliplatin-based therapy, previous adjuvant, and for initially metastatic in bivariable models (Table 2).

NLR and LMR and skin toxicity

In the univariable logistic regression model, no association was found between NLR and skin toxicity (NLR^High^ vs. NLR^Low ^OR=1.1, 95%CI: 0.4-3.2, p=0.893). This result was confirmed after adjusting for age (≥65 vs. <65 years), oxaliplatin-based chemotherapy, and number of metastatic sites (≥2 vs. <2) in a predefined multivariable logistic regression model (OR 0.7, 95%CI 0.2-2.5, p 0.585).

Similar results were found for LMR, both in univariable (LMR^High^ vs. LMR^Low^ OR=1.2, 95%CI: 0.3-4.1, p=0.810) and in the predefined multivariable (LMR^High^ vs. LMR^Low^ OR=1.9, 95%CI: 0.5-7.6, p=0.383) models.

## Discussion

The prognostic and predictive value of the NLR and LMR has been widely studied in most tumors [14-16] and with many types of treatment (chemotherapy [17], radiotherapy [18], and immunotherapy [19]). The accessibility, low cost, and ease of calculating those ratios from a simple blood count justify the growing interest in these biomarkers. Many recent studies have observed that a high NLR is associated with poor survival of patients with cancer [20]. Inflammatory cells display their role in the tumor microenvironment by influencing cancer growth and development [21]. During the inflammatory response, neutrophils may inhibit immune system activation by reducing the cytolytic activity of lymphocytes, activated T cells, and natural killer cells. This unbalance may trigger a chronic inflammatory condition leading to self-maintaining tissue damage where the growth of the tumor may depend upon a chronic inflammatory stimulus where neutrophils are persistently present [22,23]. Ultimately, these biomarkers are only the tip of the iceberg of much more complex biology concerning the complex and dynamic relationship between the tumor, its microenvironment, and the immune system of the host.

In contrast, the lymphocytes play an important role in suppressing cancer cell proliferation and migration: tumor-infiltrating lymphocytes (TILs) are associated with tumor control because they are responsible for both cellular and humoral antitumor immune responses [24]. Likewise, the monocytes associated with malignant tissue, called tumor-associated macrophages (TAM), contribute to angiogenesis and lymphangiogenesis, which lead to increased cancer cell proliferation [25].

In this retrospective longitudinal study, we have found that the early response rate is lower in patients on therapy with panitumumab and NLR^High^ and LMR^Low^. These biomarkers seem to be able to predict early response to treatment with panitumumab with good accuracy.

Our data, unfortunately, does not reach the static significance, probably due to the small sample size, but it is in accordance with data reported in the previously mentioned papers for other types of therapy.

Another interesting aspect of the multivariate analysis is the statistically significant evidence that the patients receiving panitumumab + oxaliplatin-based therapy and LMRhigh had better PFS.

We also assessed whether these biomarkers predicted the early onset of skin toxicity. Having easy-to-use and reliable biomarkers to predict the occurrence of such toxicity could help in optimizing prophylactic treatment. EGFR inhibitor-induced rash has a negative impact on the quality of life, and the release of chemokines and the recruitment of the lymphocytes are responsible for a deficient skin barrier function and, therefore, a higher risk of cutaneous bacterial infections [8]. In general, a prophylactic therapy with emollients and oral antibiotics, in particular tetracycline, is recommended [26,27] because it is well known that tetracyclines, including minocycline, have anti-inflammatory properties and reduce neutrophil chemotaxis [28,29].

In our paper, the lack of association between NLR (and LMR) and skin toxicity may depend not only on the small sample size but also on a selection bias: all patients received minocycline as prophylaxis of cutaneous toxicity starting on the first day of therapy.

Our paper has several limitations. To begin with, the small sample size of patients evaluated and, secondly, its retrospective nature leads to selection bias. It has to be acknowledged that PFS was evaluated after three to four cycles of treatment because not all patients had the same timing of radiological re-evaluation. Therefore this value is quite imprecise and is one of the limits of this retrospective work.

## Conclusions

To the best of our knowledge, this is the first study to investigate specifically the relationships between these biomarkers and panitumumab, both in terms of predicting response to treatment and comparing skin toxicity and in terms of prognosis. We have found that the basal value of NLR and LMR is predictive of early response to panitumumab treatment.

Due to the small sample size, the reported analysis should be considered exploratory and should aim at generating hypotheses to be further investigated in ad hoc designed future prospective studies.
